# The impact of mHealth interventions on health systems: a systematic review protocol

**DOI:** 10.1186/s13643-016-0387-1

**Published:** 2016-11-25

**Authors:** Jill Fortuin, Faatiema Salie, Leila H. Abdullahi, Tania S. Douglas

**Affiliations:** 1 Division of Biomedical Engineering, Department of Human Biology, Faculty of Health Sciences, University of Cape Town, Anzio Road, Observatory, Cape Town, 7925 South Africa; 2 Vaccines for Africa Initiative, Institute for Infectious Disease and Molecular Medicine, University of Cape Town, Anzio Road, Observatory, Cape Town, 7925 South Africa; 3 Division of Medical Microbiology, Department of Clinical Laboratory Services, University of Cape Town, Anzio Road, Observatory, Cape Town, 7925 South Africa

**Keywords:** mHealth, Health service, Access, Cost, Quality

## Abstract

**Background:**

Mobile health (mHealth) has been described as a health enabling tool that impacts positively on the health system in terms of improved access, quality and cost of health care. The proposed systematic review will examine the impact of mHealth on health systems by assessing access, quality and cost of health care as indicators.

**Methods:**

The systematic review will include literature from various sources including published and unpublished/grey literature. The databases to be searched include: PubMed, Cochrane Library, Google Scholar, NHS Health Technology Assessment Database and Web of Science. The reference lists of studies will be screened and conference proceedings searched for additional eligible reports. Literature to be included will have mHealth as the primary intervention. Two authors will independently screen the search output, select studies and extract data; discrepancies will be resolved by consensus and discussion with the assistance of the third author.

**Discussion:**

The systematic review will inform policy makers, investors, health professionals, technologists and engineers about the impact of mHealth in strengthening the health system. In particular, it will focus on three metrics to determine whether mHealth strengthens the health system, namely quality of, access to and cost of health care services.

Systematic review registration: PROSPERO CRD42015026070

**Electronic supplementary material:**

The online version of this article (doi:10.1186/s13643-016-0387-1) contains supplementary material, which is available to authorized users.

## Background

A health system is defined as an organization that encompasses all institutions, people and actions whose core interest is to promote, restore or maintain health [1]. It is further characterized according to its main purposes of financing, provision of inputs and service delivery/coverage. The key role players include government and consumers, and the outcomes include health, fairness in financing and responsiveness [2].

Mobile health or mHealth refers to the use of wireless communication devices to support public health and clinical practice [3]. It is considered a health enabling tool that impacts positively on the health system in terms of improved access and quality of health care and reduction in the cost of health services. Health enabling tools include health information systems, health promotion programmes, decision support systems and preventative programmes (e.g. vaccination programmes). The mobile devices or technologies used in mHealth include handsets or mobile devices, personal digital assistants (PDA) and mobile phones with PDA functions, smartphones, tablets also known as ultra-portable computers and portable media players [4].

As technology is evolving so are the capabilities of mobile phones; this has led to the widespread use of mobile phones and in turn the application of mobile health [5–7]. Globally, the number of mobile subscriptions is growing exponentially and in some countries exceeds the population size [8, 9]. The literature suggests that there is a lack of rigorous scientific evidence on the benefits of mHealth especially in terms of randomized controlled trial studies [4]. Research studies are often inconclusive due to small sample size or short study duration [10, 11]. Furthermore, there is an absence of evidence demonstrating the ability of mHealth to strengthen the health system [12].

The purpose of this systematic review is to ascertain whether mHealth has an impact on the health system, particularly on quality, access and cost. These interrelated factors are some of the biggest challenges in delivering health care globally [13] and comprise the “iron triangle” of health care [14]. To advance the performance of the health system, the components of the triangle need to be in equilibrium [15].

### Rationale

The systematic review will reveal whether mHealth solutions have an impact on improving access to health care, quality of health care and/or reducing the cost of delivering health care. It will also describe the implementation of relevant mHealth solutions, the target audiences and the outcomes. The data collected will be mapped to highlight the strengths and weakness of mHealth in terms of its impact on the health system. This will create opportunities for further research and development that results in new processes, products, publications and/or policies.

This systematic review varies from similar studies as it examines three metrics for evaluating the impact of mhealth on the health system, namely access, quality and cost. Furthermore, the study focusses on developing regions.

### Research question

The aim of this review is to give an accurate account of how mHealth impacts on the health system. This impact would be described in terms of one or more of three metrics. The research question to be addressed is: Does mHealth improve the health system in terms of access, quality and cost compared to conventional health services?

The participants in all studies assessed are human subjects. The intervention is mHealth, and the comparator is conventional health care methods. The outcome identifies if mHealth impacts the health system by assessing access, quality and/or cost of healthcare.

## Methods and design

This systematic protocol has been registered with PROSPERO, the International Database of Prospectively Register of Systematic Reviews [16]. The registration number is CRD42015026070. The authors acknowledge that it adheres to the PRISMA-P 2015 checklist as a condition of submission of systematic review protocols (see Additional file 1) [17].

## Eligibility criteria

### Study design

The type of research to be included in this review focusses on quantitative studies. The study design includes randomized and non-randomized studies. The types of non-randomized studies includes case-control, cohort and cross-sectional studies in which mHealth is the primary intervention used.

### Study participants

All studies that describe mHealth targeted at individuals or groups and aimed at that improving access to, quality of and cost of health care services will be eligible for inclusion. Study participants to be included will represent all ages, gender, ethnicity, employment status, occupation and roles in the reviewed study (i.e. patients and health professionals).

### Type of intervention

mHealth interventions to be included in the study should aim to strengthen the health system by improving access to, quality of and reducing the cost of health care.

The technology used for mHealth would include mobile devices which have cellular communication capabilities that allow for wireless interaction. The following mobile devices or mobile electronic devices, having wireless and/or 3G capabilities, will be considered as interventions for inclusion in the study: mobile phones (including android, smart and feature phones), PDA and PDA phones, tablets, ultra-portable computers and smart books [4, 18].

mHealth functions consist of voice calling, voice over internet protocol (VOIP), short message service (SMS) or text messaging, multimedia message service (MMS) and the Internet [19]. The applications that are used most often in mobile health are (1) client education and behaviour change, (2) sensors and point of care devices, (3) registries and vital event tracking, (4) data collection and reporting, (5) electronic health records, (6) electronic decision support, (7) provider-provider communication, (8) provider work planning and scheduling, (9) provider training and education, (10) human resource management, (11) supply chain management and (12) financial transactions and incentives [20].

### Outcomes

The primary outcome in this review would be to determine whether mHealth strengthens the health system. To measure the improvement, this study will extract data of concerning at least one of the three metrics with mHealth as the primary intervention. In this study, access would be defined as the prospect to acquire healthcare through the availability and supply of services [21]. Quality would refer to the delivery of healthcare which is safe, effective, timely and equitable. Most importantly, quality refers to services having a positive health outcome [22]. The cost of healthcare addresses whether there is a cost benefit, cost saving and cost effectiveness. The questions that will be used in answering the metrics include:

Does mHealth allow for patients and/or health professionals to access health services? The types of measurement that we would expect to obtain in the literature would refer to increase in patient attendance at health facilities or for services. Other measurements include patient adherence or decreases in the number of patients that are not compliant.

When using mHealth, has the quality of service been maintained or has it improved? The type of answer would include whether waiting times have been reduced, or whether the time required to access specialist care has improved because of mHealth.

What are the cost implications when using mHealth? The data to be extracted would contain information about whether mHealth has resulted in a cost benefit or cost saving.

### Study setting

The setting of the study will be limited to developing countries as defined by the United Nations Millennium Development Goals (MDG) regions. The regions include Caucasus and Central Asia, eastern Asia, Latin America and the Caribbean, northern Africa, Oceania, South-eastern Asia, southern Asia, Sub-Saharan Africa and western Asia [23].

### Search strategy

We will identify relevant studies within the period 1 January 1973 through 31 December 2016 with no language restrictions. The start date has been selected as 1 January 1973, as this is the approximate time when Motorola introduced the first cell phone [24]. The time period is broad to ensure comprehensive coverage of the literature.

The databases to be searched include: PubMed (NLM); Scopus; PsycINFO, EMBASE; Cochrane Library (Wiley) (including Cochrane Central Register of Controlled Trials (CENTRAL), Cochrane Database of Systematic Reviews, Cochrane Methodology Register, NHS Health Technology Assessment Database, ISI Web of Science (Science Citation Index), POPLINE, Global Health (Ovid), Pan-African and WHO-International Clinical Trials Registry Platform (ICTRP)).

The studies to be included will be selected using predefined search terms adapted for the databases to be used. The terms comprise both free text word and medical subject heading (MeSH). The terms will describe mHealth, health system, access, quality and cost. Table 1 depicts the search strategy to be used for PubMed, and these will be adapted as required for other databases.

**Table 1:** Preliminary

Search	Query	Items found
#6	Search (((((((((((((mhealth[All Fields]) OR telemedicine[MeSH Terms]) OR cellphone[MeSH Terms]) OR reminder system[MeSH Terms]) OR wireless technology[MeSH Terms])OR text messaging[MeSH Terms]) OR medical informatics[MeSH Terms]) OR pda[MeSH Terms]) ORsmartphone[MeSH Terms]) OR tablet computer[MeSH Terms])) AND ((((quality[MeSH Terms]) OR quality assurance[MeSH Terms]) OR clinical efficacy[MeSH Terms]) OR efficacy[MeSH Terms]))) AND ((health system[MeSH Terms]) OR health services[MeSH Terms]) Filters: Publication date from 1973/01/01 to 2015/12/31; Humans	68
#5	Search (((((((((((((mhealth[All Fields]) OR telemedicine[MeSH Terms]) OR cellphone[MeSH Terms]) OR reminder system[MeSH Terms]) OR wireless technology[MeSH Terms])OR text messaging[MeSH Terms]) OR medical informatics[MeSH Terms]) OR pda[MeSH Terms]) ORsmartphone[MeSH Terms]) OR tablet computer[MeSH Terms])) AND ((health services accessibility[MeSH Terms]) OR access[MeSH Terms]))) AND ((health system[MeSH Terms]) OR health services[MeSH Terms]) Filters: Publication date from 1973/01/01 to 2015/12/31; Humans; English	11
#4	Search (((((((((((((mhealth[All Fields]) OR telemedicine[MeSH Terms]) OR cellphone[MeSH Terms]) OR reminder system[MeSH Terms]) OR wireless technology[MeSH Terms])OR text messaging[MeSH Terms]) OR medical informatics[MeSH Terms]) OR pda[MeSH Terms]) ORsmartphone[MeSH Terms]) OR tablet computer[MeSH Terms])) AND ((((cost[MeSH Terms]) OR cost benefit[MeSH Terms]) OR cost saving[MeSH Terms]) OR cost effectiveness[MeSH Terms]))) AND ((health system[MeSH Terms]) OR health services[MeSH Terms]) Filters: Publication date from 1973/01/01 to 2015/12/31; Humans; English	19
#3	Search (((((((((((mhealth[All Fields]) OR telemedicine[MeSH Terms]) OR cellphone[MeSH Terms]) OR reminder system[MeSH Terms]) OR wireless technology[MeSH Terms])OR text messaging[MeSH Terms]) OR medical informatics[MeSH Terms]) OR pda[MeSH Terms]) ORsmartphone[MeSH Terms]) OR tablet computer[MeSH Terms])) AND ((((quality[MeSH Terms]) OR quality assurance[MeSH Terms]) OR clinical efficacy[MeSH Terms]) OR efficacy[MeSH Terms]) Filters: Publication date from 1973/01/01 to 2015/12/31; Humans; English	167
#2	Search (((((((((((mhealth[All Fields]) OR telemedicine[MeSH Terms]) OR cellphone[MeSH Terms]) OR reminder system[MeSH Terms]) OR wireless technology[MeSH Terms])OR text messaging[MeSH Terms]) OR medical informatics[MeSH Terms]) OR pda[MeSH Terms]) ORsmartphone[MeSH Terms]) OR tablet computer[MeSH Terms])) AND ((health services accessibility[MeSH Terms]) OR access[MeSH Terms]) Filters: Publication date from 1973/01/01 to 2015/12/31; Humans; English	18
#1	Search (((((((((((mhealth[All Fields]) OR telemedicine[MeSH Terms]) OR cellphone[MeSH Terms]) OR reminder system[MeSH Terms]) OR wireless technology[MeSH Terms])OR text messaging[MeSH Terms]) OR medical informatics[MeSH Terms]) OR pda[MeSH Terms]) ORsmartphone[MeSH Terms]) OR tablet computer[MeSH Terms])) AND ((((cost[MeSH Terms]) OR cost benefit[MeSH Terms]) OR cost saving[MeSH Terms]) OR cost effectiveness[MeSH Terms]) Filters: Publication date from 1973/01/01 to 2015/12/31; Humans; English	46

The search strategy was developed in consultation with a health sciences information specialist. The search strategy will be tested and revised in consultation with the information specialist prior to the final search.

### Searching other sources

The reference lists of appropriate studies will be assessed. Full text articles of the studies extracted from the reference list will be obtained and reviewed for additional information. Unpublished studies/grey literature will be identified; this is an important component of the systematic review. These studies will be sourced through: contacting the corresponding authors for missing information or unpublished data and hand searching of relevant reports and conference proceedings. Some of the databases to be searched for unpublished literature include the New York Academy of Literature, OpenGrey and the World Health Organization Working Group on mHealth.

### Study selection

Once the search strategy has been finalized and tested, the first author will retrieve all the relevant articles from the various databases. All the literature obtained will be saved in Endnote reference management software for further analysis.

The titles and abstracts of studies identified by the literature search will be screened independently by two authors for eligibility. Two authors will make a final assessment for inclusion using the full text article, and discrepancies and disagreements will be resolved by a third author. Clear reasons for exclusion will be documented by each reviewer.

### Data extraction

A standardized form will be developed and piloted to extract data from the eligible studies. Discrepancies amongst the two authors in the process of data extraction will be resolved through discussion. When no consensus is reached, a third author will mediate. Key information to be extracted includes:

- author/s and year of the study
- country of study setting
- type of facility/environment (e.g. diabetic clinic, paediatric hospital, community)
- affiliation of author
- type of participant/study population/demographic characteristics (e.g. children under 5 years attending pre-school)
- type of mobile device used
- nature of the mHealth intervention
- type of study (i.e. study design)
- type of outcomes measured
- findings/results

Data will be entered into Review Manager (RevMan) software, Version 5.1. Copenhagen: The Nordic Cochrane Centre, the Cochrane Collaboration, 2011 by JF. The second author will verify the data entered, for missing or incorrect data.

### Assessing risk of bias

Bias will be assessed by two authors independently using the criteria as stipulated by the International Cochrane Collaboration. The key elements of the criteria include randomization sequence generation, allocation concealment, blinding of participants, completeness of outcome data, selective outcome reporting and other sources of bias. For each included study, we will report our assessment of risk of bias, i.e. low, high or unclear risk for each domain, together with a descriptive summary of the information that influenced our judgement. Two authors will apply the criteria, and we will discuss any discrepancies and disagreement on bias, which will be resolved in consultation with a third author.

### Data analysis and synthesis

This systematic review will determine the impact of mHealth on the health system focussing on three metrics, namely cost, quality and access. Despite the varying participants, study designs, geographical location and intervention, the most important component is to determine the impact of mHealth on the health system using one or more of the metrics.

The result of each study will be expressed as a risk ratio with its corresponding 95% confidence interval (CI) for dichotomous data or a mean difference with its 95% CI for continuous data. The studies will be clustered around similar types of participants, interventions, study designs and outcomes for an overall estimate of effect. The data will be pooled from studies of similar interventions, participants, outcomes and study designs in a meta-analysis using the random-effects model if there is no significant statistical heterogeneity, methodological difference or high risk of bias. If variation between studies in the reported interventions, participants, study designs and outcome measures is encountered, we will not pool the results but summarize the findings in a narrative format. Regression analysis of the interrupted time series (ITS) studies will be conducted with time trends before and after the interventions. The presentation of results for the ITS study outcomes will be represented as change in level and slope [25]. In the event, ITS studies have been incorrectly analysed by the study authors and the data points have been provided, we will re-analyse them using a regression analysis.

The findings will also be mapped to depict a holistic picture of the impact of mHealth on the health system. In particular, it will highlight the benefits and challenges in the various studies analysed according to the metrics cost, quality and access.

### Dealing with missing data

When deemed necessary, the authors of studies will be contacted for all missing or incomplete data. Should there be no response from the authors, the limited data available will be included and the implications of missing/incomplete data will be discussed.

### Assessment of heterogeneity

Clinical heterogeneity will be assessed by examining types of participants, interventions and outcomes in each study. Those studies identified as being clinically homogenous will be pooled. Heterogeneity between studies will be evaluated using chi-square tests and I-squared statistics. If studies are found to be sufficiently homogenous, in terms of study population, intervention, geographical location and outcomes, the data will be pooled and estimate summary effect sizes will be determined using a fixed effects model. Alternatively, a random effects model will be used in case of heterogeneity.

### Subgroup analysis

The purpose of the subgroup analysis in this study is to determine if varying mHealth applications have an effect on the health system and in what context this occurs.

It is expected that the geographical location, mHealth intervention, health system structure and country income of various studies will be varied. Those countries with similar income levels as defined by the World Bank will be grouped [26]. Participants and mHealth interventions with similar characteristics will also be grouped.

The aim of the statistical analysis would be to determine whether the mHealth intervention has the same effect across varying subgroups, this is called a test of interaction. As we are testing more than one outcome, we are trying to explain that the mHealth intervention/common factor would yield the same positive result regardless of the characteristics such as geographical location, type of participant or structure of the health system. Conducting the subgroup analysis would add credibility to the review.

### Sensitivity analysis

A sensitivity analysis will be undertaken to determine study quality in terms of risk of bias, level of participant drop-out and statistical method (random-effect vs fixed-effect model). In addition, the sensitivity analysis will determine whether excluded studies have any impact.

## Discussion

The recent proliferation of mobile communication devices has fuelled an increase in mHealth services. It is not clear whether the promise of greater access to, improved quality and reduced cost of health care has been realized. The proposed systematic review will inform further research and innovation in terms of benefits and shortcomings that mHealth may have in the health system. The review will particularly focus on whether mHealth is effective in improving access to and quality health care services and whether costs associated with mHealth are reduced.

## Additional file

Additional file 1: PRISMA-P 2015 Checklist. (DOCX 29 kb)

## Abbreviations

MMS: Multimedia message service
PDA: Personal digital assistants
SMS: Short message service
VOIP: Voice over internet protocol
